# CT-guided biopsy in suspected spondylodiscitis: microbiological yield, impact on antimicrobial treatment, and relationship with outcome

**DOI:** 10.1007/s00256-018-2944-2

**Published:** 2018-04-16

**Authors:** Ömer Kasalak, Marjan Wouthuyzen-Bakker, Hugo J. A. Adams, Jelle Overbosch, Rudi A. J. O. Dierckx, Paul C. Jutte, Thomas C. Kwee

**Affiliations:** 1 Department of Radiology, Nuclear Medicine and Molecular Imaging, University Medical Center Groningen, University of Groningen, Hanzeplein 1, PO Box 30.001, 9700 RB Groningen, The Netherlands; 2 Department of Medical Microbiology and Infection Prevention, University Medical Center Groningen, University of Groningen, Groningen, The Netherlands; 3 Department of Radiology and Nuclear Imaging, Deventer Hospital, Deventer, The Netherlands; 4 Department of Orthopedics, University Medical Center Groningen, University of Groningen, Groningen, The Netherlands

**Keywords:** Biopsy, CT, Culture yield, Spondylodiscitis, Spine infection

## Abstract

**Purpose:**

To investigate the clinical impact of CT-guided biopsy, as performed in routine clinical practice, in patients with suspected spondylodiscitis on MRI in terms of culture yield, impact on antimicrobial treatment, and outcome.

**Methods:**

This study included 64 patients with MRI findings compatible with spondylodiscitis who underwent CT-guided biopsy.

**Results:**

Initial CT-guided biopsies were culture-positive in 20/64 (31.3%, 95% confidence interval [CI] 21.2–43.3%). Repeat CT-guided biopsies (after initial negative biopsy) were culture-positive in an additional 5/15 (33.3%, 95% CI 15.2–58.3%). Serum leukocytes, C-reactive protein, pre-biopsy use of antibiotics, neurological symptoms, MRI findings, vertebral height loss, and hyperkyphosis were not significantly different between culture-positive and culture-negative cases (*P* = 0.214–1.000); 75% (15/20) of initial CT-guided biopsies that were culture-positive provided additional information to clinicians for guiding antibiotic treatment. Sixty-two of 64 patients (96.9%, 95% CI 89.3–99.1%) would have been adequately treated if a strategy was followed that would subject all patients without clinical findings suspicious for “atypical” microorganisms and negative blood cultures to empirical antibiotics (i.e., clindamycin for coverage of Gram-positive bacteria) without using biopsy results to determine the optimal antibiotic regimen. Outcome within 6 months (development of neurologic or orthopedic complications, surgery, and death) was not significantly different (*P* = 0.751) between culture-positive and culture-negative patients.

**Conclusions:**

Although CT-guided biopsies are culture-positive in a minority of cases, the majority of positive cultures are useful to tailor antibiotic treatment. Empirical treatment with clindamycin may cover almost all micro-organisms in positive biopsy specimens, provided patients are not immunocompromised. Outcome appears similar between culture-positive and culture-negative patients.

## Introduction

Spondylodiscitis refers to disc infection with adjacent vertebral body osteomyelitis [1]. The annual incidence of spondylodiscitis in the Western world ranges from 0.4 to 2.4/100,000 [2]. Spondylodiscitis is a serious disease, causing paralysis or motor weakness in around 25% of patients, with some patients requiring surgery [1]. Furthermore, patients with initially treated spondylodiscitis have a relapse rate of spine infection of about 8% [1]. The overall attributable mortality is approximately 6% [1]. Delay in diagnosis or management is regarded as detrimental to outcome [2]. Nevertheless, the onset of spondylodiscitis-related symptoms is insidious and there is usually a delay in diagnosis of 2–6 months after the start of symptoms [1, 2]. Magnetic resonance imaging (MRI) is regarded as the imaging modality of choice for suspected spondylodiscitis [3]. Infectious Diseases Society of America (IDSA) guidelines strongly recommend to perform MRI before biopsy [4, 5]. Furthermore, there is general consensus that every effort should be made to establish a microbiological diagnosis [1, 2]. Blood cultures have been reported to be positive in approximately 58% of patients [6]. If blood cultures remain negative after 48 h, it is recommended to perform computed tomography (CT)-guided biopsy [1, 2].

Some studies indicated that the positive culture rate of CT-guided biopsies (calculated as the number of positive cultures among all biopsy specimens) is rather disappointing [7, 8], with reported percentages as low as 19.0% [8]. Recently, it was also reported that only 50% of culture-positive CT-guided biopsies provided additional information to clinicians for guiding antibiotic treatment [8]. These recent data conflict with the results of older studies, which reported positive CT-guided biopsy cultures in up to 91% [9]. One potential reason for the reported low culture yields is data contamination with spondylodiscitis mimickers (such as Modic type I degeneration, acute Schmorl node, and (osteoporotic) fractures [3]), and the inclusion of patients with a previous history of spondylodiscitis (the post-treatment MRI findings are non-specific and are known to frequently result in an erroneous diagnosis of spondylodiscitis [10]). Serious methodological biases are likely to explain the high culture yields reported in some previous studies. For example, the study that reported a 91% positive CT-guided biopsy culture yield excluded all patients with negative blood and biopsy cultures to calculate this percentage [9]. Moreover, the criteria used by clinicians for referral of patients for CT-guided biopsy were unclear in that study [9]. Therefore, further research is essential to determine both the actual positive culture rate of CT-guided biopsies and its impact on antimicrobiological treatment. Another unknown issue is whether CT-guided biopsy results have any prognostic implications in terms of progressive neurologic and orthopedic complications, the need for surgery, and mortality. If CT-guided biopsy proves to have both a low pathogen detection rate with limited treatment implications and no prognostic implications, the routine use of this invasive procedure may be reconsidered.

The purpose of this study was therefore to investigate the clinical impact of CT-guided biopsy, as performed in routine clinical practice, in patients with suspected spondylodiscitis on MRI in terms of culture yield, impact on antimicrobial treatment, and outcome.

## Materials and methods

### Study design

The local institutional review board approved this retrospective study, and waived the requirement for informed consent. The hospital’s database was searched for all patients who underwent CT-guided biopsy because of suspected spondylodiscitis between July 2008 and April 2017. Note that the protocol in our hospital dictates that MRI should be performed in all cases of suspected spondylodiscitis before biopsy is done, unless there are contra-indications for MRI. Patient inclusion criteria were: MRI findings suggestive of spondylodiscitis [3], the availability of a CT-guided biopsy, and histological and microbiological examination of the obtained tissue specimen. Patient exclusion criteria were: lack of MRI within 2 months before CT-guided biopsy, MRI findings suggestive of another condition than spondylodiscitis [3]), CT-guided biopsies that were performed because of the suspicion of a malignancy, previous history of spondylodiscitis, CT-guided aspiration of paraspinal fluid collections without any MRI findings of spondylodiscitis, and CT-guided biopsies that revealed malignancy.

### MRI acquisition and evaluation

MRI scans were performed using various clinical 1.5-T MRI systems. MRI acquisitions included sagittal and axial post-gadolinium T1-weighted sequences, sagittal and axial T2-weighted sequences, and fat-suppressed T2-weighted sequences, with slice thickness varying between 3 and 4 mm. MRI scans were re-evaluated by a radiologist (T.C.K., with 6 years of experience in musculoskeletal MRI) who was blinded to CT-guided biopsy results and follow-up data, using a PACS workstation (Carestream Vue PACS version 11.4.1.1102, Carestream Health, Inc., Rochester, NY, USA). MRI scans were reassessed to ensure that imaging findings were compatible with spondylodiscitis and to exclude other diseases such as Modic type I degeneration, acute Schmorl node, ankylosing spondylitis, SAPHO syndrome, neuropathic spine, and (osteoporotic) fractures. The MRI features for diagnosis of spondylodiscitis were used according to Hong et al. [3]; (a) typical of spondylodiscitis: involvement (T2 hyperintensity and/or contrast enhancement after gadolinium administration) of two consecutive vertebrae and the disc on MRI; or (b) atypical of spondylodiscitis: involvement of only one vertebral body, one vertebral body and one disc, two vertebral bodies without the intervening disc, and any other pattern. Presence or absence of paravertebral phlegmon (T2 hyperintense and uniformly enhancing tissue) and abscess (peripheral enhancement) was determined. Height of involved vertebrae was semi-quantitatively assessed, according to the method as described by Genant et al. [11], with > 40% height loss considered severe. Presence of hyperkyphosis of involved vertebrae, defined as a kyphosis angle greater than 40° [12, 13], was also assessed. The latter two assessments were also made for all available follow-up imaging examinations (including conventional radiography, CT, and MRI) that were performed after CT-guided biopsy. Note that 36 of 64 patients who were eventually included had follow-up MRI scans, with a median time span between biopsy and follow-up MRI of 5 months (range, 1–60 months).

### CT-guided biopsy

CT-guided biopsy of the involved level of the spine was performed by musculoskeletal-, interventional-, or neuroradiologists as part of routine clinical care. Core needle biopsy was done in all patients. Core needle size ranged between 8 and 18 gauge, depending on the preference of the attending radiologist as determined for each patient individually. The number of biopsies passes also depended on the attending radiologist’s clinical judgment and the amount of tissue obtained. Acquired tissue specimens were sent to both the pathology and microbiology department for further examination.

### Pathological and microbiological examination

One of the attending musculoskeletal pathologists evaluated the acquired biopsy specimen as part of routine clinical care, with special attention to exclude other diseases than spondylodiscitis, particularly malignancy. A medical microbiologist (M.W.-B.) interpreted the culture results as clinically relevant or suspicious of (skin) contamination. Additional serology, mycobacterial cultures, or molecular analysis were performed when clinically indicated, according to IDSA guidelines [4, 5].

### Statistical analysis

Culture yields of all initial CT-guided biopsies and repeat CT-guided biopsies (after initial negative biopsy) were calculated, along with 95% confidence intervals (CIs). Differences in serum leukocyte and C-reactive protein (CRP) levels, pre-biopsy use of antibiotics, neurological status (presence vs. absence of neurological symptoms including peripheral neuropathy and paraplegia), MRI findings (typical vs. atypical MRI findings, and presence vs. absence of paravertebral phlegmon and/or abscess, severe vertebral height loss, and hyperkyphosis) were assessed between culture-positive and culture-negative cases. The proportion of culture-positive CT-guided biopsies that provided additional information to clinicians for guiding antibiotic treatment (i.e., in those patients with negative blood cultures) was also calculated. In addition, the proportion of patients in whom standard empirical antibiotics would not cover the organism(s) that was/were detected on CT-guided biopsy was determined. Differences in outcome (defined as either development of severe vertebral height loss, hyperkyphosis, neurological deficits, or need for surgery within 6 months after CT-guided biopsy, and/or death during hospitalization) was also assessed between patients with positive and negative CT-guided biopsy cultures.

Differences between patients with positive and negative CT-guided biopsy cultures were analyzed using two-tailed unpaired *t* tests for normal distributed data, Mann–Whitney tests for non-normal distributed data, and Fisher tests for dichotomous data. Kolmogorov–Smirnov tests were first used to check whether continuous variables were normally distributed. *P* values less than 0.05 were considered statistically significant. Statistical analyses were executed using MedCalc version 17.2 Software (MedCalc, Mariakerke, Belgium).

## Results

### Patients

**Fig. 1:**
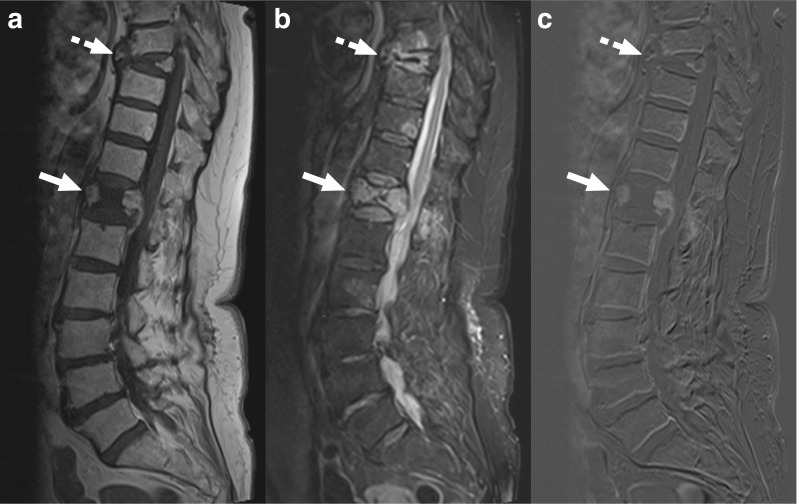
An 80-year-old woman with MRI findings consistent with osteoporotic vertebral fractures. Sagittal T1-weighted (**a**), fat-suppressed T2-weighted, and gadolinium-enhanced subtraction images (**c**) show collapse of vertebrae T12 (*continuous arrows*) and T8 (*dashed arrows*), with edema (**b**) and gadolinium enhancement of anterior and posterior portions of the T12 vertebra (**c**), and the impression of some edema in adjacent discs (**b**). However, there is no clear involvement of two consecutive vertebrae. Moreover, the involvement of multiple levels and the configuration of the affected vertebrae strongly suggest osteoporotic vertebral fractures. This patient underwent CT-guided biopsy with spondylodiscitis in the differential diagnosis of the original clinical report, but was excluded from this study

**Fig. 2:**
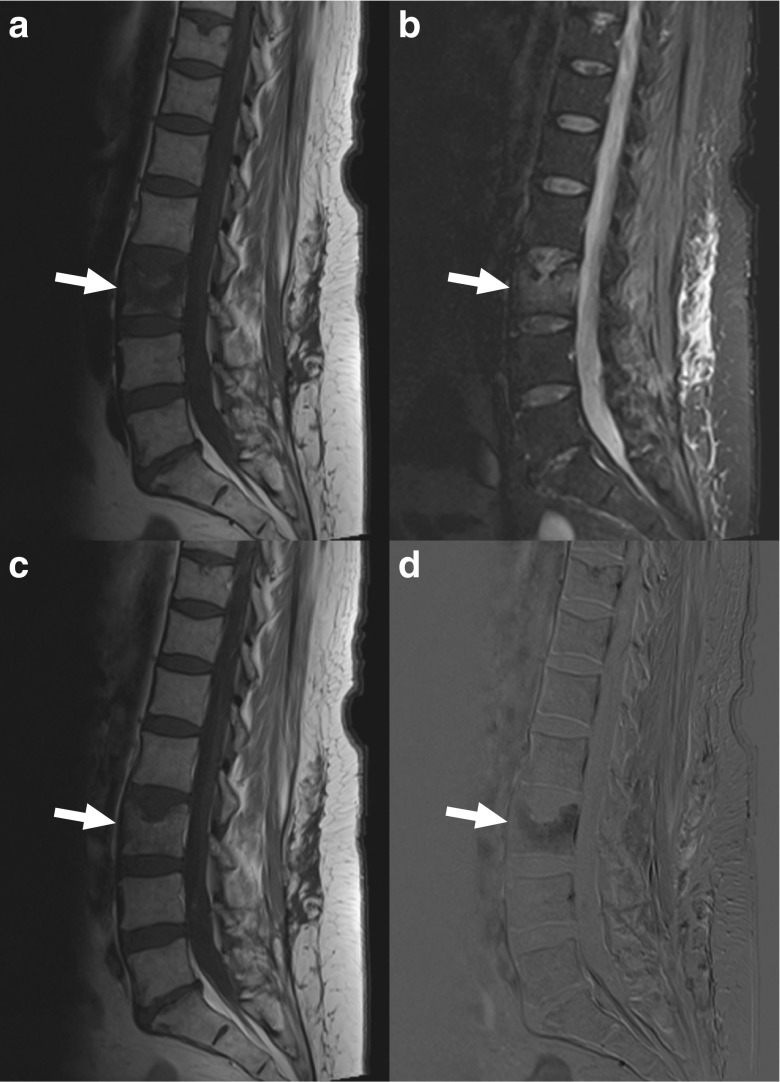
A 54-year-old woman with MRI findings consistent with acute Schmorl node. Sagittal T1-weighted (**a**), fat-suppressed T2-weighted (**b**), gadolinium-enhanced T1-weighted (**c**), and gadolinium-enhanced subtraction images (**d**) show a focal impression in the superior endplate of the L3 vertebra with surrounding pathological signal intensity (*arrows*), but involvement of only one endplate and no diffuse signal intensity abnormality of the adjacent disc. This patient underwent CT-guided biopsy with spondylodiscitis in the differential diagnosis of the original clinical report, but was excluded from this study

**Fig. 3:**
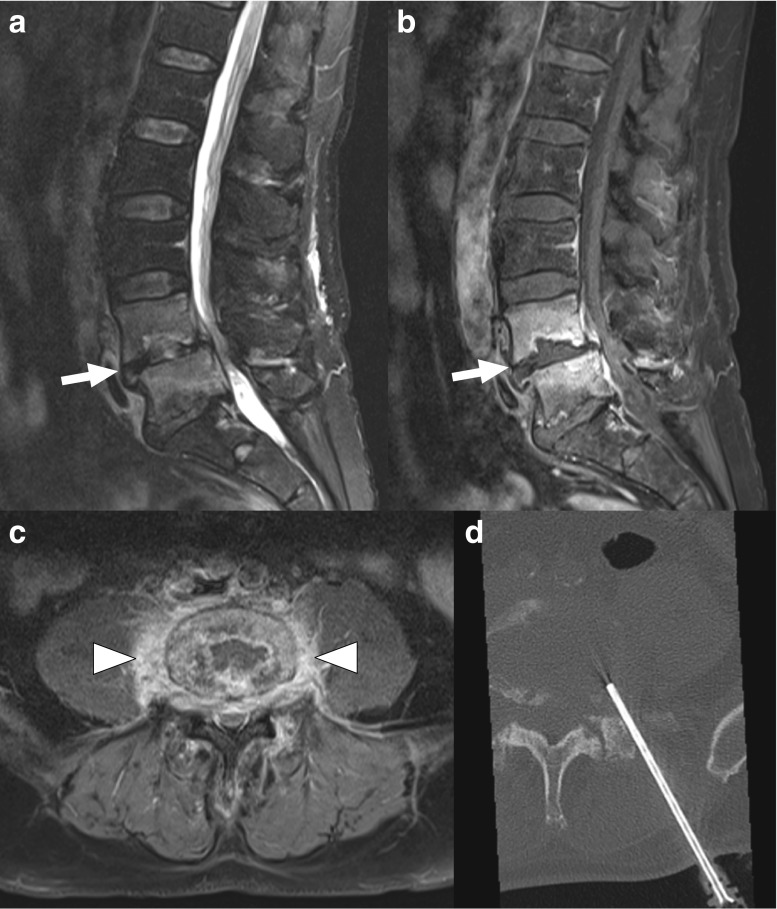
A 67-year-old man with MRI findings typical of spondylodiscitis. Sagittal fat-suppressed T2-weighted (**a**) and gadolinium-enhanced T1-weighed images (**b**) show involvement of the L4 and L5 vertebrae (*arrows*), and also increased T2 signal of the L4-L5 disc (**a**). Axial gadolinium-enhanced T1-weighted image (**c**) shows a paravertebral phlegmon (*arrowheads*). CT-guided biopsy was performed (**d**), with positive cultures for *Staphylococcus warneri*

**Fig. 4:**
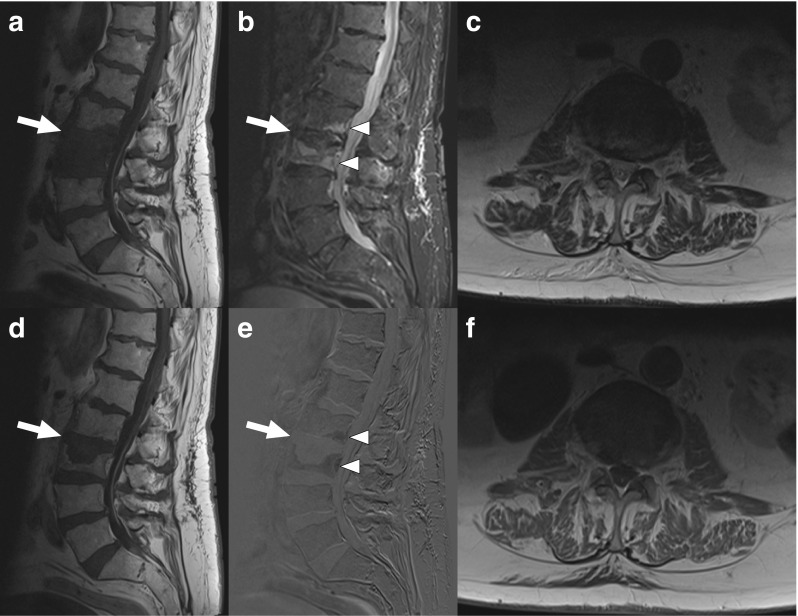
A 88-year-old man with MRI findings atypical of spondylodiscitis. Sagittal T1-weighted (**a**), fat-suppressed T2-weighted (**b**), gadolinium-enhanced T1-weighted (**d**), and gadolinium-enhanced subtraction images (**e**) show pathological signals in both the L2 and L3 vertebrae (*arrows* and *arrowheads*), but no clear T2 hyperintensity or gadolinium enhancement of the L2–L3 disc. Axial T2-weighted (**c**) and gadolinium-enhanced T1-weighted images (**f**) do not show any paravertebral phlegmon or abscess either. CT-guided biopsy was culture-negative

A total of 84 patients were potentially eligible for inclusion. Of these 84 patients, eight were excluded because of previous history of spondylodiscitis, four were excluded because of MRI findings suggestive of another diagnosis than spondylodiscitis (osteoporotic fractures [*n* = 2], acute Schmorl node [*n* = 1], and a paravertebral mass of unknown origin that was stable over the past 12 years [*n* = 1]) (Figs. 1 and 2)), three were excluded because no MRI was performed to establish the diagnosis, two were excluded because of both lack of recent (< 2 months) MRI and previous history of spondylodiscitis, two were excluded because the obtained CT-guided biopsy specimens were erroneously not sent to the microbiological laboratory, and one was excluded because of CT-guided aspiration of a paraspinal fluid collection without any MRI findings of spondylodiscitis. Thus, 64 patients (34 male and 30 female patients, with mean age of 61.7 ± standard deviation [SD] ± 16.5 years, range, 16–91 years) were finally included. The spondylodiscitis foci were localized in the cervical (7.8%, 5/64), thoracic (28.1%, 18/64), thoracolumbar (4.7%, 3/64), lumbar (42.2%, 27/64), and lumbosacral (17.2%, 11/64) spine. Involvement of one, two, three, and four vertebral-disc complexes was seen in 87.5% (56/64), 9.4% (6/64), 1.6% (1/64), and 1.6% (1/64) patients, respectively. Sixteen patients (25%) had undergone previous spinal surgery. Representative MRI images are shown in Figs. 3 and 4.

### Microbiological yield CT-guided biopsies

Twenty of 64 initial CT-guided biopsies (31.3%, 95% CI 21.2–43.3%) were culture-positive. From the 44 culture-negative cases, a second CT-guided biopsy was performed in 15 cases, from which an additional five were culture-positive (33.3%, 95% CI 15.2–58.3%). The isolated microorganisms of both initial and additionally obtained biopsies are displayed in Table 1. None of the biopsies showed any signs of malignancy on histopathological examination. Arterial bleeding occurred during one CT-guided biopsy, which was successfully managed conservatively. No complications occurred during the other 78 CT-guided biopsies. Table 1 shows the microorganisms that were detected in the positive cultures. There were no statistically significant differences in leukocytes, CRP levels, use of antibiotics, neurological symptoms, presence of typical MRI findings of spondylodiscitis, paravertebral pleghmon/abscess, severe vertebral height loss, and hyperkyphosis before biopsy between patients with positive and negative CT-guided biopsy cultures (*P* = 0.214–1.000) (Table 2).

**Table 1:** Isolated microorganisms

Positive initial CT-guided biopsy cultures (*n* = 20)	Positive repeated CT-guided biopsy cultures (*n* = 5)	Positive blood cultures (*n* = 9)^a,b^
*Propionibacterium acnes* (*n* = 7) *Staphylococcus aureus* (*n* = 3) *Enterococcus faecalis* (*n* = 2) *Candida albicans* (*n* = 1) *Candida crusei* (*n* = 1) *Escherichia coli* and *Bacteroides* species (*n* = 1) *Staphylococcus saccharolyticus* (*n* = 1) *Streptococcus agalactiae* (*n* = 1) *Streptococcus salivarius*, *Streptococcus* species, and *Neisseria* species (*n* = 1) *Streptococcus* species (*n* = 1) *Streptococcus sanguinis* (*n* = 1)	*Staphylococcus aureus* (*n* = 2), *Proprionibacterium acnes* (*n* = 1) *Staphylococcus saprophyticus* (*n* = 1) *Staphylococcus warneri* (*n* = 1)	*Staphylococcus aureus* (*n* = 3) *Enterococcus faecalis* (*n* = 2) *Bacteroides* species (*n* = 1) *Staphylococcus epidermidis* (*n* = 1) *Streptococcus gallolyticus* (*n* = 1) *Parvimonas micra* (*n* = 1)

^a^Five positive blood cultures were also culture-positive on CT-guided biopsy (all initial CT-guided biopsies), all for the same microorganism (*Staphylococcus aureus* [*n* = 3] and *Enterococcus faecalis* [*n* = 2])

^b^Four positive blood cultures (*Bacteroides* species [*n* = 1], *Staphylococcus epidermidis* [*n* = 1], *Streptococcus gallolyticus* [*n* = 1], and *Parvimonas micra* [*n* = 1]) were culture-negative on CT-guided biopsy

**Table 2:** Comparison of laboratory, clinical, and imaging features between patients with positive and negative CT-guided biopsy cultures

Parameter	Positive CT-guided biopsy cultures (*n* = 25)	Negative CT-guided biopsy cultures (*n* = 39)	*P* value
Leukocytes (10^9^/l)	9.9 ± 5.8^a, p^	9.6 ± 4.3^a,q^	0.801^b^
CRP (mg/ll)	73.8 ± 72.3^a,p^	62.8 ± 66.6^a,r^	0.555^b^
Pre-biopsy use of antibiotics	4/25 (16.0%, 95% CI 6.4–34.7%)	6/39 (15.4%, 95% CI 7.3–29.7%)	1.000^c^
Neurological symptoms	3/25 (12.0%, 95% CI 4.2–30.0%)	2/39 (5.1%, 95% CI 1.4–16.0%)	0.371^c^
Typical MRI findings of spondylodiscitis	22/25 (88.0%, 95% CI 70.0–95.8%)	28/39 (71.8%, 95% CI 56.2–83.5%)	0.214^c^
Paravertebral phlegmon and/or abscess	18/25 (72.0%, 95% CI 52.4–85.7%)	27/39 (69.2%, 95% CI 53.6–81.4%)	1.000^c^
Severe vertebral height loss*	3/25 (12.0%, 95% CI 4.2–30.0%)	1/39 (2.6%, 95% CI 0–13.2%)	0.291^c^
Hyperkyphosis**	0/25 (0%, 95% CI 0–13.2%)	1/39 (2.6%, 95% CI 0–13.2%)	1.000^c^
Poor outcome***	6/25 (24.0%, 95% CI 11.5–43.4%)	7/39 (18.0%, 95% CI 9.0–32.7%)^s^	0.751^c^

^a^Mean ± standard deviation (SD)

^b^Two-tailed unpaired *t* test

^c^Fisher’s exact test

^d^Median with interquartile range between parentheses

^e^Mann–Whitney test

^p^Two missing data

^q^Six missing data

^r^Five missing data

^s^One missing data

^t^Seven patients with negative cultures were not treated with antibiotics after CT-guided biopsy; none of them experienced a poor outcome. After exclusion of these seven patients, there was still no significant difference in outcome between culture-positive and culture-negative patients (*P* = 1.000)

* > 40% vertebral height reduction

** Kyphosis angle greater than 40°

*** Development of new > 40% vertebral height reduction, hyperkyphosis, neurological deficits, or need for surgery within 6 months after CT-guided biopsy, and/or death during hospitalization

### Impact of positive biopsy cultures on antimicrobial treatment

In 46 of 64 biopsied patients (71.9%) blood cultures were also obtained, which were positive in nine cases (19.6%, 95% CI 10.7–33.2%). The isolated microorganisms are depicted in Table 1. Five of these latter nine cases were also culture-positive on CT-guided biopsy (all for the same microorganism) whereas four cases had positive blood cultures but negative biopsy. Therefore, 75% (15/20) of initial CT-guided biopsies that were culture-positive and 23.4% (15/64) of all biopsies performed provided additional information to clinicians for guiding antibiotic treatment. When considering both culture-positive positive initial and culture-positive repeat CT-guided biopsies, these proportions reached 80% (20/25) and 25.3% (20/79), respectively.

Assuming a standard empirical treatment for Gram-positive bacteria with clindamycin, the microorganisms in the biopsy specimens of 16 of 20 patients with positive biopsies but negative blood cultures would have been adequately covered (i.e., positive biopsy results did not alter this empirical treatment). Of the four remaining patients with positive biopsy cultures, two were immunocompromised (of whom one presented with concomitant intestinal symptoms), which would have alerted the clinicians that empirical treatment without (positive) culture testing might not be adequate (*Candida crusei*, and a combination *Escherichia coli* and *Bacteroides pyogenes* were detected in these two patients). The two other patients with positive biopsy cultures would not have been covered with empirical antibiotics and had no clinical signs to suggest “atypical” microorganisms (*Candida albicans*, and a combination of *Streptococcus salivarius*, *Streptococcus species*, and *Neisseria species* were detected in these two patients). Thus, 62 of the entire population of 64 patients (96.9%, 95% CI 89.3–99.1%) would have been adequately treated if a strategy was followed that would subject all patients without clinical findings suspicious for “atypical” microorganisms and negative blood cultures to empirical antibiotics without using biopsy results to determine the optimal antibiotic regimen.

### Association with outcome

Thirteen of 64 patients (28.3%) had a poor outcome (four patients developed severe vertebral collapse, three patients required spinal surgery, two patients died during hospitalization (the exact causes of death remained unclear), two patients developed severe vertebral collapse and hyperkyphosis, one patient developed paraplegia due to a new epidural abscess (which could not be surgically treated due to the patient’s poor condition), and one patient developed severe vertebral collapse and hyperkyphosis and underwent spinal surgery). However, there was no significant difference in outcome (*P* = 0.751) between patients with positive CT-guided biopsy cultures (6/25 patients with poor outcome) and negative CT-guided biopsy cultures (7/39 patients with poor outcome) (Table 2).

## Discussion

The results of this study show that CT-guided biopsy in patients with MRI findings compatible with spondylodiscitis is culture-positive in about one-third of patients, and that this number is comparable for additional biopsies in primary culture-negative cases. The biopsy yield percentages in this study may be more realistic than previously reported numbers that suffered from methodological flaws [7–9, 14], although local antibiotic resistance could change yields between institutions. The same applies to the investigation on the association of several laboratory, clinical (including the pre-biopsy use of antibiotics), and MRI findings with CT-guided biopsy results, with none of them being statistically associated with a positive culture. Positive CT-guided biopsies yielded additional information to clinicians for guiding antimicrobial treatment in 75% of patients. These data seem to support the routine use of CT-guided biopsy in patients with suspected spondylodiscitis and negative blood cultures, in line with general consensus [1, 2, 4, 5]. On the other hand, among the entire population, all CT-guided biopsies were useful to tailor antibiotic treatment in only 25% of patients. Moreover, the present study found no significant difference in outcome between biopsy culture-positive and culture-negative patients. A recent study by Kim et al. [15] reported no significant difference (*P* = 0.157) in treatment success (defined as survival and absence of signs of infection at the end of the therapy) either between 75 patients with microbiologically confirmed spondylodiscitis (whether by means of blood or biopsy cultures) and 76 patients without microbiologically confirmed spondylodiscitis. Given the above-mentioned numbers, and the fact that (despite being relatively safe, with transient complications in 2% [16]) CT-guided biopsy is costly, invasive, and uses potentially harmful ionizing radiation, the idea has been coined to manage patients without clinical findings suspicious for “atypical” pathogens with a course of empirical antibiotic therapy, without performing CT-guided biopsy [8]. Our data also showed that more than 95% of patients would be adequately covered with antibiotics with such a strategy. Potential drawbacks of such an approach, however, are the risk antibiotic resistance development and that it is less likely to be effective in regions in which antimicrobial resistance is already a major problem. Although further research on this topic is necessary, it appears justifiable to consider such a strategy when CT-guided biopsy is relatively contra-indicated, for example in patients with hemorrhagic diatheses, in those who are anticipated to have problems lying still during the procedure or require general anesthesia, and in settings in which there is no physician available with expertise in CT-guided biopsy of the spine. A future randomized prospective study, in which an algorithm of clinical symptom criteria → blood culture and imaging criteria → biopsy → treatment will be compared to an alternative algorithm of clinical symptom criteria → blood culture and imaging → treatment, would be of value to gain more insight into the utility of CT-guided biopsies in spondylodiscitis. Importantly, immunocompromised patients should be excluded from such a study because these patients may present with (opportunistic) micro-organisms that are not covered by empirical antibiotics.

The present study had several limitations. First, due to its retrospective design, there may have been selection bias. Second, in the absence of a positive culture, there is no good reference standard for spondylodiscitis. Although all MRI scans were reviewed to exclude spondylodiscitis mimickers, MRI does not reach 100% specificity [3], and it cannot be excluded that non-spondylodiscitis cases were included. Third, blood cultures were obtained in only 71.9% of patients. Fourth, there was a lack of data to determine the effect of core needle size, aspiration, and number of passes on quality and volume of the biopsy specimen. Fifth, exact data on type and duration of antibiotics use could not be retrieved, and their effect on culture yields could not be assessed. Sixth, clinical outcome was defined as loss of vertebral body stature, degree of kyphosis, neurological deficits, need for surgery within 6 months, and death. However, these outcomes are likely multifactorial, and this analysis was not corrected for effects of relevant variables such as degree of antibiotic resistance, medical history, age, and pre-existing spinal degenerative joint disease.

In conclusion, although CT-guided biopsies are culture-positive in a minority of cases, the majority of positive cultures are useful to tailor antibiotic treatment. Empirical treatment with clindamycin may cover almost all micro-organisms in positive biopsy specimens, provided patients are not immunocompromised. Outcome appears similar between biopsy culture-positive and culture-negative patients.
